# Hybrid treatment of penetrating trauma of the thoraco-abdominal aorta with bullet embolism to the popliteal artery - case report

**DOI:** 10.1590/1677-5449.202500962

**Published:** 2026-01-09

**Authors:** Thiago Filomena Lombard, Luisa Silveira Birck

**Affiliations:** 1 Instituto de Saúde São Lucas de Pato Branco – ISSAL, Pato Branco, PR, Brasil.

**Keywords:** gunshot wound, trauma, aorta, ballistic embolism, case report

## Abstract

Bullet embolism is a rare complication of gunshot wounds, but its incidence has increased due to the increasing number of wounds caused by low-velocity firearms in civilian settings. The increase in urban violence and widespread use of firearms with low-velocity projectiles have made diagnosis of ballistic embolism more common, so it is essential to recognize the risk factors for such events. Both treatment of the entry wound and removal of the ballistic embolism can be successfully performed by open, endovascular, or hybrid surgery, and the particularities of each case should be considered. We report the case of a 16-year-old male patient who presented with penetrating aortic trauma in addition to an intra-arterial firearm projectile in the right popliteal artery and underwent hybrid surgery to treat his injuries.

## INTRODUCTION

Bullet embolism is a rare complication of gunshot wounds. It can occur when the projectile has low kinetic energy when it penetrates a blood vessel or the heart and passes through one of the walls, without exiting through another wall. Additionally, the diameter of the vessel involved must be larger than the projectile.^1,2^

While this is a rare phenomenon, the increasing incidence of low velocity gunshot wounds in civilian settings has raised the probability of occurrence.^1^

The rarity of bullet embolism and its tendency not to provoke immediate symptoms can lead to delayed diagnosis and treatment, with risks of limb loss and death, whether due to the original injury or because of migration.^3^

This article reports a case of bullet embolism with entry via the thoracoabdominal aorta and embolization to the right popliteal artery, treated successfully with a hybrid approach.

The protocol was approved by the Research Ethics Committee at our institution (opinion number 7.231.289; Ethics Appraisal Submission Certificate number 80193724.0.0000.5330).

## CASE REPORT

A 16-year-old, previously healthy, male patient was taken to the emergency room with a gunshot wound to the right thoracoabdominal transition. On admission, he underwent computed tomography with contrast, showing pulmonary contusion and bilateral hemothorax, in addition to retroperitoneal hemorrhage, and free fluid in the pelvis.

**Figure 1:**
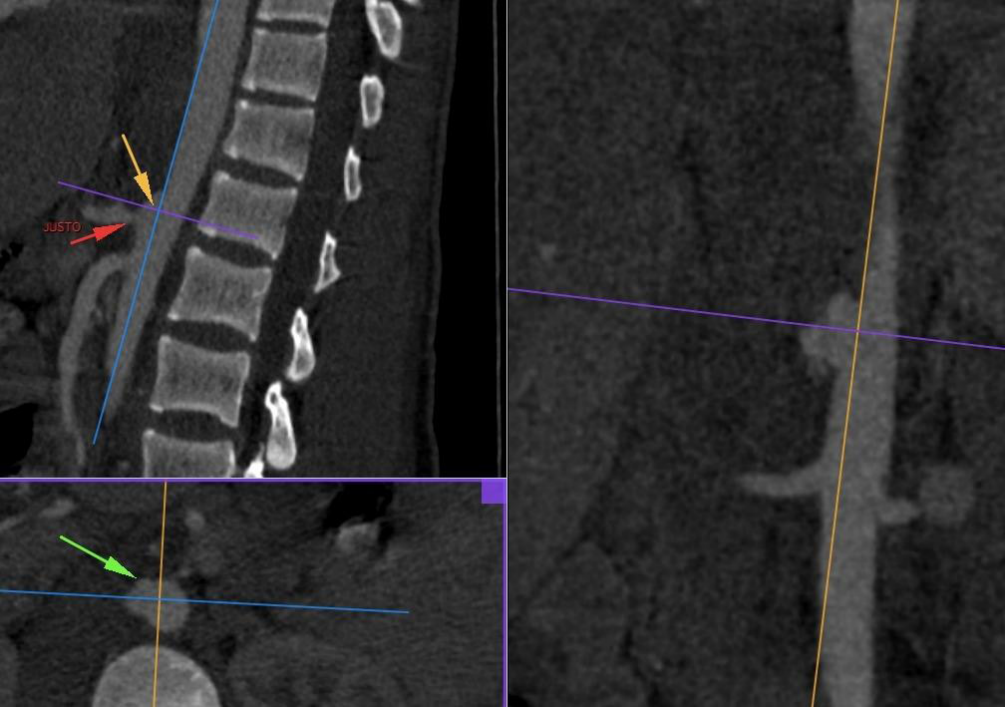
Computed tomography angiography images showing an aortic pseudoaneurysm at the thoracoabdominal transition, adjacent to the origin of the celiac trunk.

**Figure 2:**
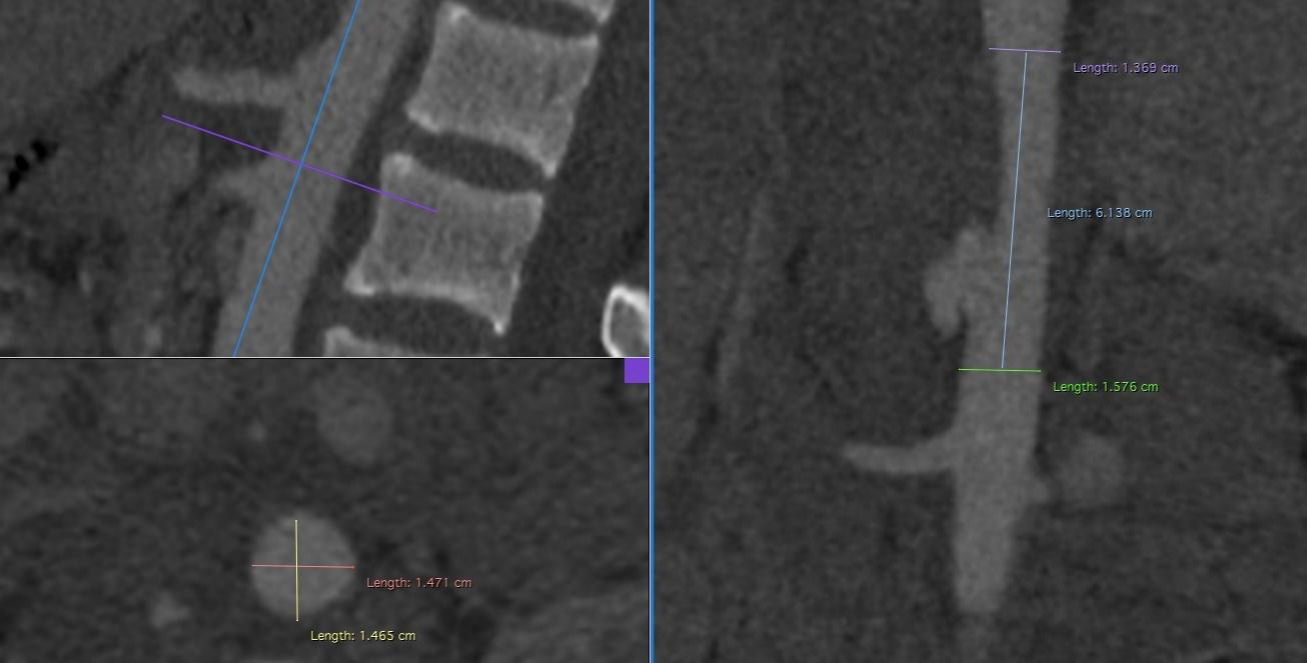
Computed tomography angiography images used to plan surgery, with measurements of the diameters of the aorta for endoprosthesis positioning.

**Figure 3:**
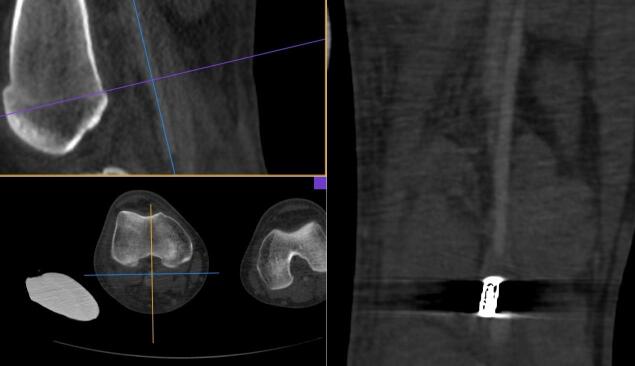
Computed tomography angiography images showing the bullet inside the arterial lumen, in the topography of the P2 segment of the popliteal artery.

The patient underwent explorative laparotomy at the first hospital and no injuries to major vessels or the viscera were found. A thoracostomy was performed and a chest tube with water seal was fitted. Four days later, he complained of intense pain and paresis of the right lower limb. Arterial Doppler ultrasonography showed occlusion of the right popliteal artery by a bullet lodged inside the vascular lumen. The patient was taken for angiotomography, which showed a grade II penetrating aortic trauma (pseudoaneurysm) at the thoracoabdominal transition, adjacent to the origin of the celiac trunk (Figures 1 and 2), and an intra-arterial bullet in segment P2 of the right popliteal artery (Figure 3).

The patient was admitted to our vascular surgery service hemodynamically stable, with no chest or abdominal pains. The right lower limb was cold, popliteal and distal pulses were absent, and the limb pain and paresis were still present.

**Figure 4:**
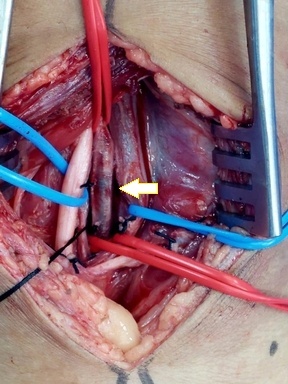
Dissection of the structures of the popliteal fossa. The arrow shows the popliteal artery at the center of the image. The intraluminal bullet can be seen due to transparency.

**Figure 5:**
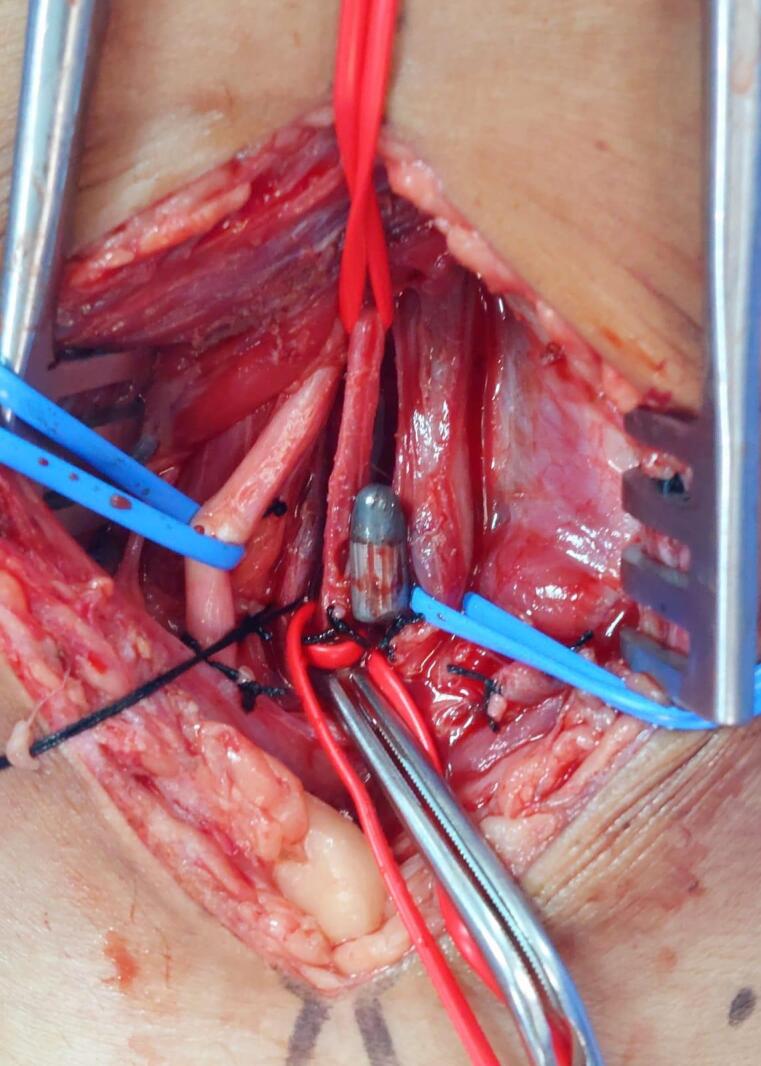
Removal of the bullet from within the popliteal artery lumen.

**Figure 6:**
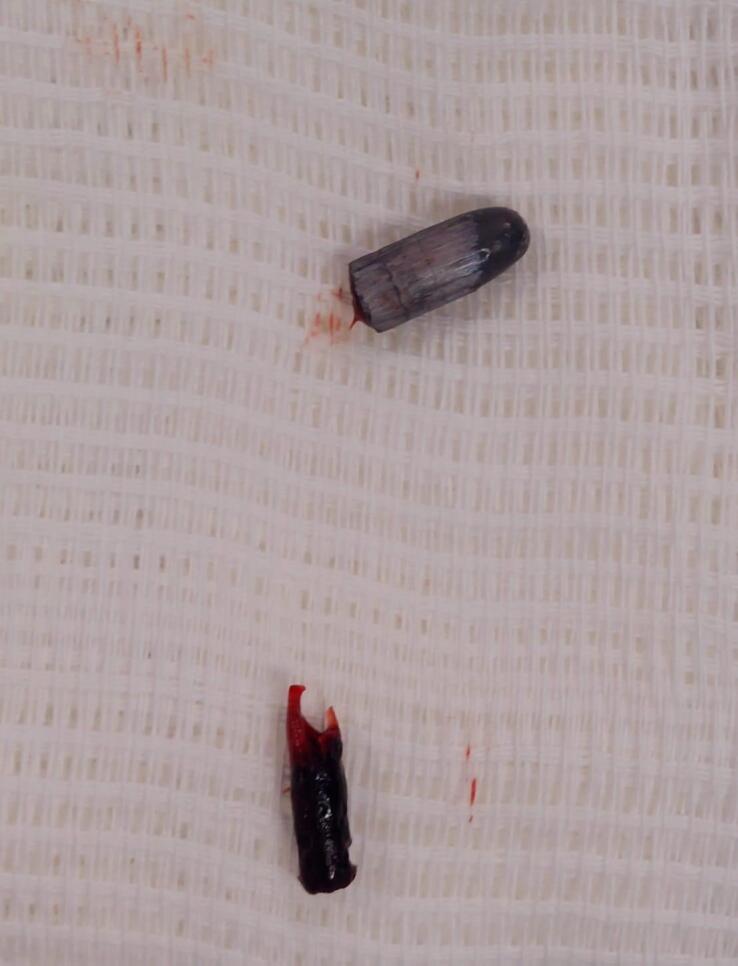
Bullet removed from the popliteal artery, together with the thrombus formed distal to the site at which it lodged.

**Figure 7:**
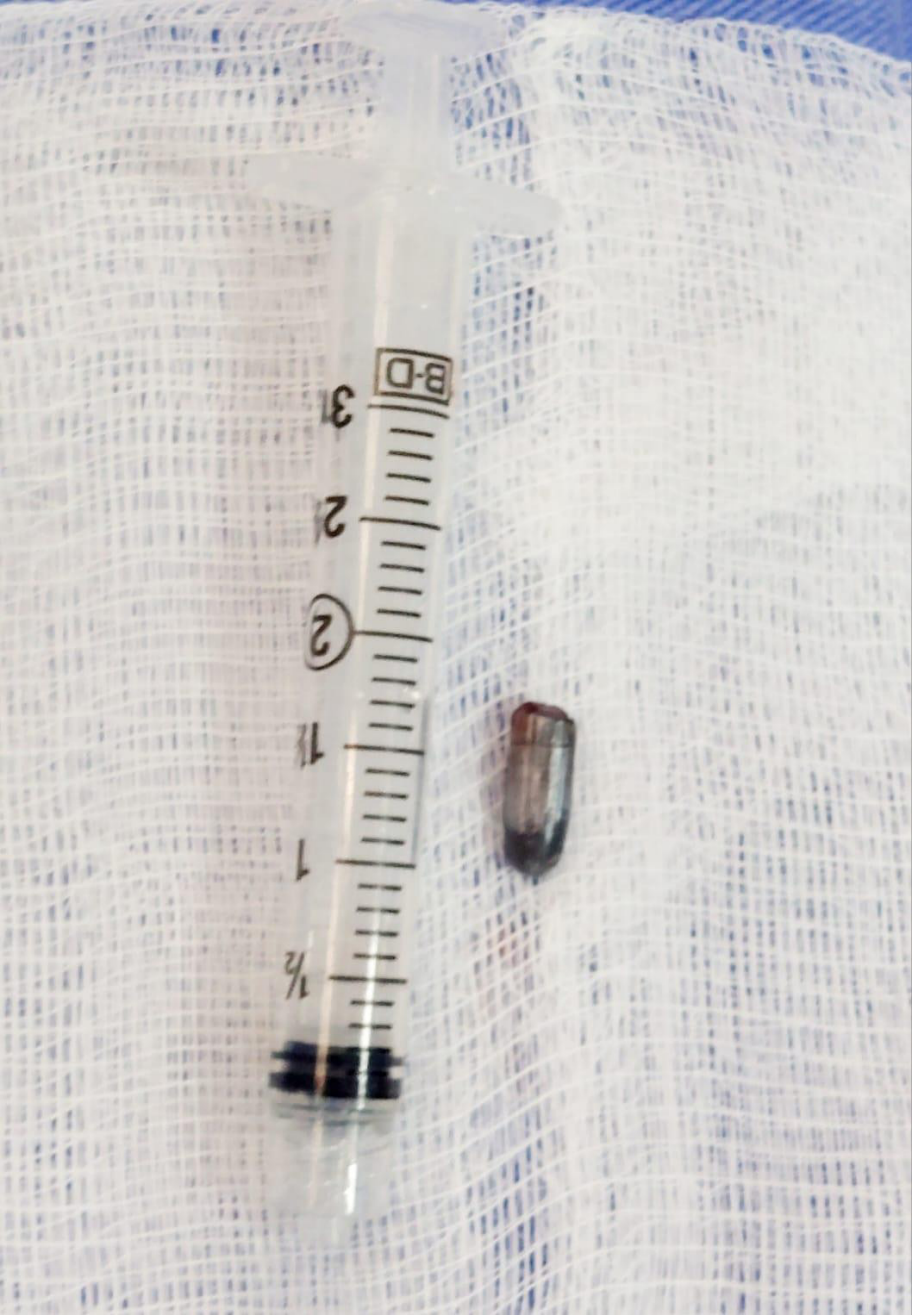
Illustration of the size of the bullet removed from the popliteal artery.

Endovascular repair of the penetrating injury to the thoracoabdominal aorta was performed using the iliac branch of an abdominal endoprosthesis measuring 16 x 16 x 82 mm, via a femoral access, because the diameters of the aorta were 14.2 mm (anteroposterior) x 14.0 mm (transverse). The distance from the aortic injury to the origin of the superior mesenteric artery was 3.3 cm and it was necessary to cover the celiac trunk origin because of its proximity to the pseudoaneurysm. Next, the patient was placed in ventral decubitus and underwent arteriotomy of the popliteal artery via a posterior approach, the bullet was removed, and flow was reestablished (Figures 4, 5, 6, and 7).

The patient recovered well postoperatively, with hemodynamic stability and improvement of symptoms in the right lower limb. A physical examination conducted in the immediate postoperative period identified the pedal pulse and peripheral perfusion was good. There were no hemodynamic repercussions in the territory fed by the celiac trunk, no changes to hepatic transaminase levels, and no signs of visceral ischemia. The patient was discharged from the vascular surgery service on the third postoperative day.

**Figure 8:**
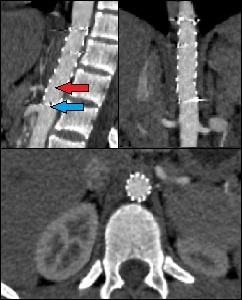
Follow-up computed tomography angiography 2 months after endovascular repair of the aortic pseudoaneurysm. It is possible to observe the wound has sealed and the celiac trunk is covered by the endoprosthesis, with flow maintained inside it from collaterals (red arrow). Note that the superior mesenteric artery is patent (blue arrow).

Follow-up computed tomography angiography was conducted 60 days after the procedure, showing the aortic injury sealed. The origin of the celiac trunk had been covered by the endoprosthesis, but there was flow inside the trunk, supplied by collaterals. The superior mesenteric artery remained patent (Figure 8). The patient was asymptomatic, with relation both to the thoracoabdominal region and to the right lower limb.

## DISCUSSION

Bullet embolism is a rare event. Studies estimate incidence of 0.3% during the Vietnam war and 1.1% during the conflicts in Afghanistan and Iraq.^4,5^ Low energy gunshots, which are more common in civilian settings, involve a higher risk of incomplete penetration of blood vessels and, consequently, of embolization. The incidence of bullet emboli in non-military settings is not yet known.^5^

For a bullet embolism to occur, the projectile must only have sufficient energy to penetrate one of the vessel walls, remaining within the lumen, without exiting the vessel. In civil traumas, 80% of cases are caused by small caliber and low velocity bullets.^2^ Once inside the vessel, the direction of embolization will depend on hydrostatic pressure, on the severity of the patient’s condition, and on the position they were in at the time of wounding, in addition to anatomic factors.^6^

The majority of patients are males (91%) aged from 20 to 40 years.^5^ Statistically, multiple gunshot wounds increase the probability of bullet embolism; however, it is more likely that a patient will present the phenomenon secondary to a single gunshot wound, since this is the most common type of firearm injury in general.^5^ The patient in the case reported herein is younger than the mean age reported in the literature (he was 16 years old) and had suffered a single gunshot wound.

Wounds that result in bullet embolism are generally located in the chest (39%), followed by the abdomen (23%), and cervical region (11%). In patients with wounds to the trunk (chest, abdomen, and pelvis), bullet embolization occurred more frequently in cases with anterior entry sites (54.3%).^5^

Older studies showed that bullet embolism was twice as common in the arterial system.^1^ However, a more recent systematic review^5^ showed a higher frequency of bullet embolic events involving venous injuries (56%), followed by arterial (27%), and cardiac (15%) involvement. One of the hypotheses the authors ventured to explain this change was the increased use of noninvasive imaging methods, enabling more asymptomatic embolic events to be detected. With regard to arterial injuries, the most common artery of entry was the aorta (38%), followed by the carotid (21%), femoral (12%), and iliac (10%) arteries.^5^

Half of all embolized bullets lodge in the lower limbs, following the direction of blood flow (anterograde). It is important to note that the bullet material reaches the cerebrovascular system in more than 1/4 of cases involving the arterial system.^5,7^ While the embolic event described in this report involved the right lower limb, studies indicate that the incidence of emboli is twice as high in the left leg, possibly because of the asymmetry of the aortic bifurcation.^1^ Arterial bullet emboli are symptomatic in the majority of cases (65 to 70%).^7^

Suspicion of bullet emboli should be heightened in the following scenarios: 1) discrepancy between the number of entry and exit wounds; 2) clinical findings that are not compatible with the visible injuries, such as neurological changes in distant regions; 3) limbs suddenly presenting pallor, coldness, and weakened pulses, suggesting acute ischemia; 4) discrepancies between clinical and radiological findings, when imaging exams show the bullet in a location that is not compatible with its apparent trajectory.

Additional signs described include hemodynamic instability associated with a penetrating thoracic wound, absence of an exit wound, radiographic changes such as the bullet appearing in different positions in successive scans, presence of the bullet within the silhouette of the heart or in an unexpected location, and absence of the bullet along its presumed trajectory. Important later manifestations include intermittent claudication, ischemic pain, gangrene, pericardial hemorrhage, arrhythmia, sepsis, and pseudoaneurysm formation.^7,8^

Endovascular treatments are well-established in certain scenarios, such as for blunt thoracic aorta traumas and nowadays are increasingly being employed for penetrating traumas.^9^

In the case reported herein, it was decided to treat the entry wound to the thoracoabdominal aorta with endovascular repair. Conventional surgical treatment of thoracic aortic injuries requires thoracotomy and substitution of the thoracic aorta with prosthetic grafts. With endovascular treatment, an endoprosthesis can be fitted during a less invasive procedure, with reduced blood loss, and no need to clamp the aorta, with faster patient recovery. Brito et al. reported reduced risk of postoperative complications such as myocardial infarction, sepsis, extended time on mechanical ventilation, and acute respiratory distress syndrome, in patients with traumatic injuries to the aorta treated with endovascular methods.^10^

In view of the proximity of the wound to the origin of the celiac trunk, it was decided to cover the celiac origin. This enables the distal sealing zone to be extended in endovascular repair of aortic diseases, making it possible to treat a larger number of patients with endoprostheses. Delle et al. recommend endovascular treatment of aneurysmal disease of the thoracic aorta with intentional coverage of the celiac trunk in selected cases. In some situations, there may be transitory elevation of hepatic enzymes, indicating borderline liver perfusion and underscoring the importance of preoperative planning.^11^ In the present case, the patient showed no signs of visceral ischemia and there was no elevation of hepatic enzyme levels.

Treatment options for removal of bullet emboli include surgical extraction, endovascular capture, and hybrid approaches.^4,12,13^ The majority of authors agree that bullets embolized in the arterial system, particularly when symptomatic, should be removed as soon as they are identified, to minimize the risk of permanent ischemic sequelae.^4,5^ Indications for removal of asymptomatic arterial emboli include risk of further distal embolization, clot formation, and potential for arterial occlusion.^2,14^

The decision may be taken not to remove the bullet if the embolus has already caused visceral or neurological infarction and occurrence of additional damage is unlikely, if the risks of the procedure outweigh the potential benefits of recovery, or in cases of asymptomatic emboli in portal and pulmonary circulation.^7^

Generally, the recommended treatment is removal of the projectile, preferably by arteriotomy with visual inspection of the site, rather than catheter embolectomy, due to the risk of intimal damage.^2^

The success rate of endovascular removal attempts was approximately 63% and this was the method most frequently used for venous system injuries. Open surgical exploration was necessary to remove the embolus in 57% of cases because of the size of the bullet.^5^ Endovascular extraction can reduce post-procedural complications because it has a substantial success rate.^5,15^

In the case reported herein, there were no endovascular devices available to extract the projectile. Moreover, the site where the foreign body had lodged facilitated surgical exploration and it was removed successfully via arteriotomy of the popliteal artery.

## Data Availability

Todos os dados gerados ou analisados estão incluídos neste artigo e/ou no material suplementar.
