# Supplementary material for: Hybrid treatment of penetrating trauma of the thoraco-abdominal aorta with bullet embolism to the popliteal artery - case report
Source: J Vasc Bras. 2026 Jan 9;24:e20250096. doi: 10.1590/1677-5449.202500962 (PMC12822376; doi:10.1590/1677-5449.202500962)
Supplement: Translation

# Translation: Tratamento híbrido de trauma penetrante de aorta toracoabdominal com embolização balística para artéria poplítea - relato de caso

**DOI:** 10.1590/1677-5449.202500961

**Authors:** Thiago Filomena Lombard, Luisa Silveira Birck

**Affiliations:** 1 Instituto de Saúde São Lucas de Pato Branco – ISSAL, Pato Branco, PR, Brasil.

**Keywords:** ferimento arma de fogo, trauma, aorta, embolia balística, relato de caso

## Abstract

A embolia balística é uma complicação rara decorrente de ferimentos por arma de fogo, cuja incidência tem aumentado devido ao crescente número de lesões provocadas por projéteis de baixa velocidade no contexto civil. O aumento da violência urbana e o uso disseminado de armas de fogo com esse tipo de projétil tornam o diagnóstico de embolia balística mais frequente, tornando essencial o reconhecimento dos fatores de risco associados a esse evento. Tanto o tratamento do ferimento de entrada quanto a remoção do êmbolo balístico podem ser realizados com sucesso por via cirúrgica aberta, endovascular ou híbrida, devendo ser consideradas as particularidades de cada caso. Relatamos o caso de um paciente do sexo masculino, de 16 anos, que apresentou trauma penetrante de aorta, além da presença de projétil intra-arterial na artéria poplítea direita, submetido a abordagem híbrida para o tratamento das lesões.

## INTRODUÇÃO

A embolia balística é uma complicação incomum após ferimentos por arma de fogo. Para que ocorra, o projétil deve apresentar baixa energia cinética no momento em que penetra o vaso sanguíneo ou o coração, atravessando apenas uma das paredes, sem transfixá-las. Além disso, o diâmetro do vaso deve ser maior que o do projétil^1,2^.

Apesar de se tratar de um fenômeno raro, o aumento na incidência de ferimentos por arma de fogo de baixa velocidade no contexto civil aumenta a probabilidade de sua ocorrência^1^.

A raridade e a frequente falta de sintomas precoces relacionados à embolia balística podem levar ao atraso no diagnóstico e no tratamento, resultando em risco de perda de membro e de morte, tanto pela lesão inicial quanto pelo evento de migração^3^.

No presente artigo, relatamos um caso de embolia balística com sítio de entrada na aorta toracoabdominal e embolização para a artéria poplítea direita, tratada com sucesso por abordagem híbrida.

O protocolo foi aprovado pelo Comitê de Ética em Pesquisa de nossa instituição (parecer número 7.231.289; Certificado de Apresentação para Apreciação Ética: 80193724.0.0000.5330).

## RELATO DO CASO

Paciente do sexo masculino, 16 anos, previamente hígido, foi encaminhado ao setor de emergência devido a ferimento por arma de fogo em transição toracoabdominal direita. Na admissão, foi realizada tomografia computadorizada com contraste, que evidenciou contusão pulmonar e hemotórax bilateral, além de hemorragia retroperitoneal e líquido livre na pelve.

O paciente foi submetido a laparotomia exploradora no hospital de origem, sem identificação de lesões de grandes vasos ou vísceras, sendo realizada toracostomia em selo d'água à direita. Quatro dias após, apresentou dor intensa e paresia do membro inferior direito. Foi realizado eco-Doppler arterial, que identificou oclusão da artéria poplítea direita por projétil de arma de fogo localizado no interior do lúmen vascular. Em seguida, o paciente foi submetido a angiotomografia, que revelou trauma penetrante aórtico grau III (pseudoaneurisma) na transição toracoabdominal, adjacente à emergência do tronco celíaco (Figuras 1 e 2), e projétil de arma de fogo intra-arterial em segmento P2 da artéria poplítea direita (Figura 3).

**Figura 1:** Imagem de angiotomografia computadorizada demonstrando pseudoaneurisma de aorta na transição toracoabdominal, adjacente à emergência do tronco celíaco.

**Figura 2:** Imagens de angiotomografia computadorizada demonstrando o planejamento cirúrgico, com mensuração dos diâmetros da aorta para o posicionamento da endoprótese.

**Figura 3:** Imagem de angiotomografia computadorizada demonstrando projetil no interior do lúmen arterial, na topografia do segmento P2 da artéria poplítea.

O paciente foi admitido em nosso serviço de Cirurgia Vascular hemodinamicamente estável, sem dor torácica ou abdominal. O membro inferior direito apresentava temperatura reduzida, ausência dos pulsos poplíteo e distais, além de dor e paresia.

Foi realizada correção endovascular da lesão penetrante da aorta toracoabdominal utilizando ramo ilíaco de endoprótese abdominal medindo 16 x 16 x 82 mm, por acesso femoral, considerando que o diâmetro da aorta era de 14,2 mm (anteroposterior) x 14,0 mm (laterolateral). A distância entre a lesão aórtica e a origem da artéria mesentérica superior era de 3,3 cm, sendo necessária a cobertura do tronco celíaco devido à proximidade do pseudoaneurisma. Em seguida, o paciente foi posicionado em decúbito ventral e submetido a arteriotomia de artéria poplítea por abordagem posterior, com retirada do projétil de arma de fogo e restabelecimento do fluxo (Figuras 4, 5, 6 e 7).

**Figura 4:** Dissecção das estruturas da fossa poplítea. A seta indica a artéria poplítea no centro da imagem, sendo possível observar o projetil intraluminal por transparência.

**Figura 5:** Retirada do projétil do interior do lúmen da artéria poplítea.

**Figura 6:** Projetil removido da artéria poplítea, acompanhado de trombo em formação distalmente ao local de impactação.

**Figura 7:** Demonstração do tamanho do projétil removido da artéria poplítea.

O paciente apresentou boa evolução pós-operatória, mantendo estabilidade hemodinâmica e melhora sintomática do membro inferior direito. No exame físico do pós-operatório imediato, apresentou pulso pedioso e boa perfusão periférica. Não houve repercussões hemodinâmicas no território irrigado pelo tronco celíaco, com evolução sem alterações nas transaminases hepáticas e sem sinais de isquemia visceral. O paciente recebeu alta do serviço de Cirurgia Vascular no 3º dia pós-operatório.

Foi realizada angiotomografia computadorizada de seguimento 60 dias após o procedimento, observando-se selamento da lesão aórtica. A origem do tronco celíaco encontrava-se coberta pela endoprótese, porém foi detectado fluxo em seu interior, proveniente de colaterais. A artéria mesentérica superior permanecia pérvia (Figura 8). O paciente estava assintomático tanto do ponto de vista toracoabdominal quanto em relação ao membro inferior direito.

**Figura 8:** Angiotomografia computadorizada de seguimento, realizada 2 meses após a correção endovascular do pseudoaneurisma aórtico. Observa-se selamento da lesão, com cobertura do tronco celíaco pela endoprótese, mantendo fluxo em seu interior por meio de colaterais (seta vermelha). Nota-se perviedade da artéria mesentérica superior (seta azul).

## DISCUSSÃO

A embolia balística é um evento raro. Estudos estimam uma incidência de 0,3% durante a Guerra do Vietnã e 1,1% nos conflitos no Afeganistão e Iraque^4,5^. Projéteis de baixa energia, mais comuns no ambiente civil, implicam risco aumentado de penetração incompleta dos vasos sanguíneos e, consequentemente, de embolização. A incidência de êmbolos balísticos em contextos não militares ainda não é totalmente conhecida^5^.

O projétil deve ter energia suficiente apenas para penetrar uma das paredes do vaso e permanecer no interior de seu lúmen, sem transfixá-lo. No trauma civil, 80% dos casos são decorrentes de projéteis de pequeno calibre e baixa velocidade^2^. Uma vez no interior do vaso, a direção da embolização dependerá da pressão hidrostática, da gravidade e da posição em que o indivíduo se encontrava no momento do ferimento, além de fatores anatômicos^6^.

A maioria dos pacientes são do sexo masculino (91%), com idade entre 20 e 40 anos^5^. Estatisticamente, múltiplos ferimentos por arma de fogo aumentam a probabilidade de embolia balística; entretanto, o mais provável é que o paciente apresente o fenômeno secundário a um único ferimento, pois esta é a forma mais comum de lesão por arma de fogo em geral^5^. No caso apresentado, o paciente é mais jovem do que a média relatada na literatura (16 anos) e apresentava somente um único ferimento por arma de fogo.

Os ferimentos que resultam em embolia balística estão geralmente localizados no tórax (39%), seguidos pelo abdome (23%) e pela região cervical (11%). Nos ferimentos ocorridos no tronco (tórax, abdome e pelve), a embolização do projétil ocorreu mais frequentemente em casos com entrada anterior (54,3%)^5^.

Estudos mais antigos apontavam que a embolia balística ocorria até duas vezes mais no sistema arterial^1^. Porém, em revisão sistemática mais recente^5^, verificou-se maior frequência de eventos embólicos de projéteis resultantes de lesão venosa (56%), seguida por arterial (27%) e cardíaca (15%). Uma das hipóteses levantadas pelos autores para essa mudança foi o aumento do uso de métodos de imagem não invasivos, permitindo maior detecção de eventos embólicos assintomáticos. Quanto aos ferimentos arteriais, o sítio de entrada mais comum foi a aorta (38%), seguida pelas artérias carótida (21%), femoral (12%) e ilíaca (10%)^5^.

Metade dos projéteis embolizados impacta nas artérias dos membros inferiores, seguindo a direção do fluxo sanguíneo (anterógrada). É importante destacar que, em mais de 1/4 dos casos envolvendo o sistema arterial, o material balístico alcança o sistema cerebrovascular^5,7^. Embora o evento embólico apresentado neste relato tenha ocorrido no membro inferior direito, estudos indicam que a incidência de embolia na perna esquerda é duas vezes maior, possivelmente devido à assimetria da bifurcação aórtica^1^. Os êmbolos balísticos arteriais são sintomáticos na maioria dos casos (65 a 70%)^7^.

Deve-se aumentar a suspeita de embolia por projétil de arma de fogo nas seguintes situações: 1) discrepância entre o número de ferimentos de entrada e de saída; 2) achados clínicos não compatíveis com as lesões visíveis, como déficits neurológicos em topografias distantes; 3) membro subitamente apresentando palidez, frialdade e redução de pulsos, sugerindo isquemia aguda; 4) dissociação clínico-radiológica, quando o exame de imagem demonstra o projétil em localização não condizente com o trajeto aparente.

Sinais adicionais descritos incluem instabilidade hemodinâmica associada a ferimento torácico penetrante, ausência de orifício de saída, alterações radiográficas como mudança de posição do projétil em controles sucessivos, presença do projétil dentro da silhueta cardíaca ou em local inesperado, bem como ausência de projétil em regiões do trajeto presumível. Entre as manifestações tardias, destacam-se claudicação intermitente, dor isquêmica, gangrena, derrame pericárdico, arritmias, sepse e formação de pseudoaneurismas^7,8^.

Os tratamentos endovasculares estão bem estabelecidos em determinados cenários, como nos traumatismos contusos da aorta torácica, e, atualmente, vêm sendo empregados com frequência crescente em traumatismos penetrantes^9^.

No caso relatado, optou-se pela correção endovascular do ferimento de entrada na aorta toracoabdominal. O tratamento cirúrgico convencional das lesões da aorta torácica requer toracotomia e substituição da aorta torácica por prótese. Com o tratamento endovascular, a endoprótese pode ser implantada por meio de procedimento menos invasivo, com menor perda sanguínea, sem necessidade de clampeamento da aorta e com recuperação mais rápida do paciente. Brito et al. relataram menor risco de complicações pós-operatórias, como infarto do miocárdio, sepse, tempo de ventilação mecânica prolongado e síndrome do desconforto respiratório agudo, em pacientes com lesões traumáticas na aorta tratados pelo método endovascular^10^.

Devido à proximidade da lesão com a origem do tronco celíaco, optou-se por sua cobertura. A cobertura da origem do tronco celíaco permite estender a zona de selamento distal para o reparo endovascular de doenças aórticas, possibilitando o tratamento de um maior número de pacientes com endoprótese. Delle et al. apoiam o tratamento endovascular da doença aneurismática da aorta torácica com cobertura intencional do tronco celíaco em casos selecionados. Em algumas situações, pode ocorrer elevação transitória das enzimas hepáticas, indicando perfusão limítrofe do fígado e reforçando a importância do planejamento pré-operatório^11^. No presente relato, o paciente evoluiu sem sinais de isquemia visceral e sem elevação de enzimas hepáticas.

As opções terapêuticas para a remoção dos êmbolos balísticos incluem extração cirúrgica, captura endovascular ou abordagem híbrida^4,12,13^. A maioria dos autores concorda que projéteis embolizados para o sistema arterial, especialmente os sintomáticos, devem ser removidos assim que forem reconhecidos, a fim de minimizar o risco de sequelas isquêmicas permanentes^4,5^. As indicações para a remoção de êmbolos arteriais assintomáticos incluem risco de nova embolização distal, formação de coágulos e oclusão arterial potencial^2,14^.

Pode-se optar pela não remoção quando o êmbolo balístico já tiver causado infarto visceral ou neurológico e for improvável a ocorrência de dano adicional, ou quando os riscos do procedimento superarem os benefícios potenciais da recuperação, além dos casos de embolia assintomática nas circulações portal e pulmonar^7^.

Geralmente, o tratamento preconizado é a remoção do projétil, preferencialmente por arteriotomia com inspeção visual da íntima no local, em vez de embolectomia por cateter, devido ao risco de lesão intimal^2^.

A taxa de sucesso das tentativas de remoção endovascular foi de aproximadamente 63%, sendo essa abordagem mais frequentemente empregada no sistema venoso. A exploração cirúrgica aberta foi necessária em 57% dos casos para a recuperação do êmbolo, devido ao seu tamanho^5^. A extração endovascular pode reduzir as complicações do procedimento, uma vez que apresenta taxa de sucesso substancial^5,15^.

No presente relato, não havia dispositivos endovasculares disponíveis para a extração do projétil. Além disso, o sítio de impactação do corpo estranho mostrou-se favorável à exploração cirúrgica, sendo realizada a remoção por meio de arteriotomia da artéria poplítea, com sucesso.

## Data Availability

All of the data generated or analyzed are included in this article and/or the supplementary material.
